# The New Rubber Suit

**Published:** 1878-10

**Authors:** 


*The New Rubber Suit*—We are informed by Dr. R. Finley Hunt, is progressing well, and presents a satisfactory appearance so far. The case will be in the regular course before the Courts at Washington, in October next. The main points on which the Cummings' Patent seems to rest its validity are controverted in this new case, and apparently successfully. It is hoped that the matter can be now speedily brought to a final issue. One of the most hopeful signs of the case is, that the Bacon people wish to postpone a hearing of it. H.

